# Cardio-metabolic abnormalities among patients with severe mental illness at a Regional Referral Hospital in southwestern Uganda

**DOI:** 10.1371/journal.pone.0235956

**Published:** 2020-07-17

**Authors:** David Collins Agaba, Richard Migisha, Godfrey Katamba, Scholastic Ashaba

**Affiliations:** 1 Department of Physiology, Mbarara University of Science and Technology, Mbarara, Uganda; 2 Department of Physiology, King Ceasor University, Kampala, Uganda; 3 Department of Psychiatry, Mbarara University of Science and Technology, Mbarara, Uganda; 4 Department of Psychiatry, Kamapala International University, Kampala, Uganda; Beijing Key Laboratory of Diabetes Prevention and Research, CHINA

## Abstract

Patients with severe mental illness (SMI) have a higher burden of premature cardio-metabolic abnormalities, including diabetes mellitus, hypertension, hyperlipidemia, and obesity resulting into a 3-fold increase in mortality, and up to 20% reduction in life expectancy compared to the general population. Although over 30% of Ugandans have some form of mental illness, there are no national or hospital-based screening guidelines for cardio-metabolic abnormalities among these patients a general trend in most low-income countries. The screening rates for cardio-metabolic abnormalities in most low-income countries are at only 0.6%. The objective of this study was to describe the cardio-metabolic abnormalities among patients with SMI at Mbarara Regional Referral Hospital. Through a cross-sectional study, we recruited 304 patients with SMI and evaluated them for cardio-metabolic abnormalities using the National Cholesterol Education Programme Adult Treatment Panel III criteria for dyslipidemias, World Health Organisation criteria for diabetes mellitus, obesity, and the Joint national committee criteria for hypertension. We then determined the proportion of participants who met the criteria for each of the individual cardio-metabolic abnormalities. Of the 304 participants, 44.41% were male and 55.59% female with a mean age of 38.56±13.66 years. Almost half (46.38%) of the participants were either overweight or obese, 33.22% had abdominal obesity, 40.46% were hypertensive, 34.11% had low high-density lipoproteins, 37.42% had hypertriglyceridemia and 34.77% had hypercholesterolemia. Based on fasting blood sugar, 11.18% and 9.87% had pre-diabetes and diabetes respectively. There is a high level of cardio-metabolic abnormalities among patients with psychiatric disorders and thus metabolic screening for these abnormalities should be done routinely during psychiatric reviews. There is a need for national guidelines for screening of metabolic abnormalities among patients with SMI so that these abnormalities can be detected early enough at stages where they can be either reversed or delayed to progress to cardiovascular disease.

## Introduction

Patients with severe mental illness (SMI) have a higher burden of premature cardio-metabolic abnormalities, including diabetes mellitus, hypertension, hyperlipidemia, and obesity compared to the general population. As a result, these patients tend to have up to a 3-fold increase in mortality and a reduction in life expectancy [1, 2]. In fact, patients with SMI die on average 20–30 years earlier than their counterparts without SMI [3] and it has been established that 50–75% of patients with SMI die as a result of cardiovascular disease [4].

Two meta-analyses of systematic metabolic monitoring of patients with schizophrenia and bipolar disorder found that about one in two (50%) had obesity, one in three (33%) had diabetes mellitus or prediabetes, two in five (40%) had hypertriglyceridemia, and two in five (40%) had hypertension [5, 6]. This high prevalence of cardio-metabolic abnormalities is linked to deleterious lifestyles common among patients with SMI such as unhealthy dietary choices, excessive alcohol consumption, smoking and reduced physical activity [7] in addition to obesogenic side effects of antipsychotic medication [8].

There is a lot of variation in the rates of cardio-metabolic screening in people with SMI [9, 10], with high screening rates observed in high-income countries such as United States of America [11] but very low rates of screening of about only 0.6% in low-income countries [12]. The low screening rates result in late diagnosis of these abnormalities at stages where lifestyle modification approaches are no longer very beneficial, thus leading to progression to cardiovascular disease.

Although over 30% of Ugandans have some form of mental illness [13], there are no national or hospital-based screening guidelines for cardio-metabolic abnormalities among patients with SMI. Thus far, data on cardio-metabolic abnormalities among patients with SMI in Uganda are limited. The aim of this study was to describe the cardio-metabolic abnormalities among patients with SMI attending the outpatient mental health clinic at Mbarara Regional Referral Hospital (MRRH). Documenting the burden of cardio-metabolic abnormalities among patients with SMI is key, so as to inform policy makers to consider formulation of guidelines for screening of metabolic abnormalities. Routine and systematic cardio-metabolic screenining will enable initiation of appropriate management of cardio-metabolic abnormalities in a timely manner, in this patient population and this may contribute towards reduced mortality and improved quality of life. Moreover, there is epidemiological evidence to suggest that routine cardio-metabolic screening could avert premature cardio-metabolic disease among patients with SMI [14].

## Materials and methods

This study was part of a bigger cross-sectional study that was conducted at the outpatient mental health clinic of MRRH between October 2018 and March 2019 to identify the prevalence and associated factors of metabolic syndrome among patients with SMI [15]. MRRH is a public health facility in southwestern Uganda that serves about five million people, mainly from 10 catchment districts of southwestern Uganda. It also serves as a teaching hospital for Mbarara University of Science and Technology (MUST) Medical school. It is located 270 kilometers from Kampala the capital city.

The outpatient mental health clinic is run at the psychiatric ward twice every week, registering an average of 100 patients per clinic and on average about 1200 new patients per year. The clinic is run by a team of three psychiatrists, three occupational therapists, one counselor, two social workers, three psychiatric clinical officers, five psychiatric nurses, and postgraduate students.

We consecutively recruited all patients with DSM-V confirmed diagnosis of severe mental illness, both men and women, aged 18 years and above, in remission phase or having recovered from an acute episode who attended the clinic during the study period. We excluded all patients who had acute symptoms of mental illness, those who had no insight and those who were considered unable to consent by the attending clinician. We also excluded pregnant women as pregnancy would affect the interpretation of waist circumference and could potentially confound blood pressure findings in case of pregnancy-induced hypertension. Patients below 18 years of age were not enrolled for the study since their cardio-metabolic risk is generally very low.

We recruited participants consecutively as they came for review at the mental health clinic as long as they met the inclusion criteria until the sample size was obtained. The sample size of 304 was computed using the Kish Leslie formula of 1965 [16] using 1.96 as the critical value at 5% level of significance, taking the prevalence of metabolic syndrome among patients with severe mental illness in sub-Saharan Africa to be 23.2%, based on a study from South Africa [17], 0.05 as a margin of error and adjusting for a non-response rate of 10%. Participants who were eligible for the study were given comprehensive information regarding the nature and purpose of the study and a consent document was read to them in the local language or English depending on the language they understood by a trained research assistant. They were given a chance to ask questions for clarity before they provided consent. Those who accepted to participate were given a consent form to sign before enrollement in the study.

The study variables of interest included age, weight, height, abdominal circumference, blood pressure, fasting blood glucose, fasting triglyceride level (TG), total cholesterol, and high-density lipoprotein (HDL). Other variables included sex, marital status, residence (urban or rural), education level, history of smoking, history of alcohol use, psychiatric diagnosis, type of psychotropic medication and duration on psychotropic medication. The outcome variables were obesity, hypertension, diabetes mellitus, and dyslipidemia (hypertriglyceridemia, hypercholesterolemia, and low high-density lipoproteins). Data were collected by research assistants trained to handle data with confidentiality.

### Measurements

Socio-demographic and clinical data were captured using a structured questionnaire. Sociodemographic and clinical data included age, sex, marital status, level of education, tribe, religion, employment, residence, alcohol intake (history of or currently taking alcohol), smoking status (history of or currently smoking), level of physical activity (inadequate if < 150 min of moderate exercise or 75min of vigorous/ week and adequate if ≥150 min of moderate exercise or 75min of vigorous /week), weight, height, DSM IV diagnosis, duration of mental illness, type of psychotropic medication, and duration of psychotropic medication. Weight was measured using a calibrated secca weighing scale (secca 762, GmbH and Co. KG, Hamburg, Germany) to the nearest 0.5 kg with the participant not wearing shoes and heavy clothes. Height was taken using a stadiometer to the nearest 0.5 cm with the participant standing upright with the heel, buttock, and upper back along the same vertical plane. Waist circumference was measured in cm using a non-stretchable measuring tape in the horizontal plane midway between the inferior margin of the ribs and the superior border of the iliac crest, at the end of normal expiration. Blood pressure was measured on the left arm in mmHg by auscultation method using a calibrated sphygmomanometer and a stethoscope with the participant seated on a chair with his or her back supported, feet on the floor, arm supported, and cubital fossa at heart level after 5 minutes of rest. Three readings were measured at 5-minute interval taking the first and fifth Korotkoff sounds as the systolic and diastolic blood pressure (DBP) respectively. The average of the last two systolic and diastolic measurements was taken as the mean SBP and DBP respectively [18].

### Laboratory tests

The laboratory tests were fasting blood sugar and fasting lipid profile. Fasting blood sugar in mmol/l was measured using a Freestyle Optium Exceed glucometer after at least 8 hours of fasting using capillary blood obtained by finger prick. Fasting lipid profile was tested using three mls (3mls) of venous blood drawn from the cubital fossa after at least 8 hours of fasting. Those who had not fasted for the 8 hours were only included if they were willing to wait at the clinic till they completed the 8 hours fast. The samples were analyzed for total cholesterol, high-density lipoprotein (HDL), triglycerides (TGs) and low-density lipoprotein (LDL) using an ELITechGroup PIT-CHSL-4-v21 (09/2016) machine following the enzymatic-colorimetric method. All procedures were done following standard operating procedure.

Pre-diabetes was defined as fasting capillary whole blood concentration ≥6.1 mmol/l but less than 7.0 mmol/l. Diabetes mellitus was defined as fasting capillary whole blood glucose concentration ≥7.0 mmol/l or currently on medication for diabetes mellitus [19, 20]. Hypertension was defined as systolic blood pressure level ≥130 mmHg and/or diastolic blood pressure level ≥80 mmHg and/or taking medications for high blood pressure [18]. Body mass index (BMI) was computed by dividing the weight (kg) by the height in meters squared (m^2^) and used to develop categories of underweight, normal weight, overweight and obesity. Overweight was defined as BMI 25.0- <30kg/m^2^ and obesity as BMI ≥30kg/m^2^ [21]. Abdominal obesity was defined as a waist circumference ≥102 cm in males and ≥88 cm in females [21]. Dyslipidemia was defined as total cholesterol ≥200mg/dl, High-Density Lipoprotein (HDL) <40 mg/dl in males and <50mg/dl in females or triglycerides ≥150 mg/dl according to the National Cholesterol Education Programme Adult Treatment Panel III (NCEP ATP III) criteria [22]. We then determined the proportion of participants who met each of the above definitions to be able to describe the cardio-metabolic abnormalities.

### Data handling and analysis

The data were cleaned and then double entered into Epi Data 3.1, after which they were exported to STATA version 12 (Stata Corp, College Station, Texas, USA) for analysis. Descriptive analysis of all variables was done. Continuous variables were described as mean±SD while categorical variables were summarized as proportions and percentages. Categorical and continuous variables were compared based on sex using the chi-square test and Student *t-*test respectively.

### Ethics

Ethical clearance for the study was obtained from the Mbarara University of Science and Technology Research Ethics Committee (No. 07/08-18) and the Uganda National Council for Science and Technology (HS 2548). We respected the guidelines of Helsinki and CIOMS-2002 (Council for International Organizations of Medical Sciences) regarding research with humans, avoiding any type of physical or moral damage. Written informed consent was obtained by research assistants from all participants.

## Results

**Fig 1:**
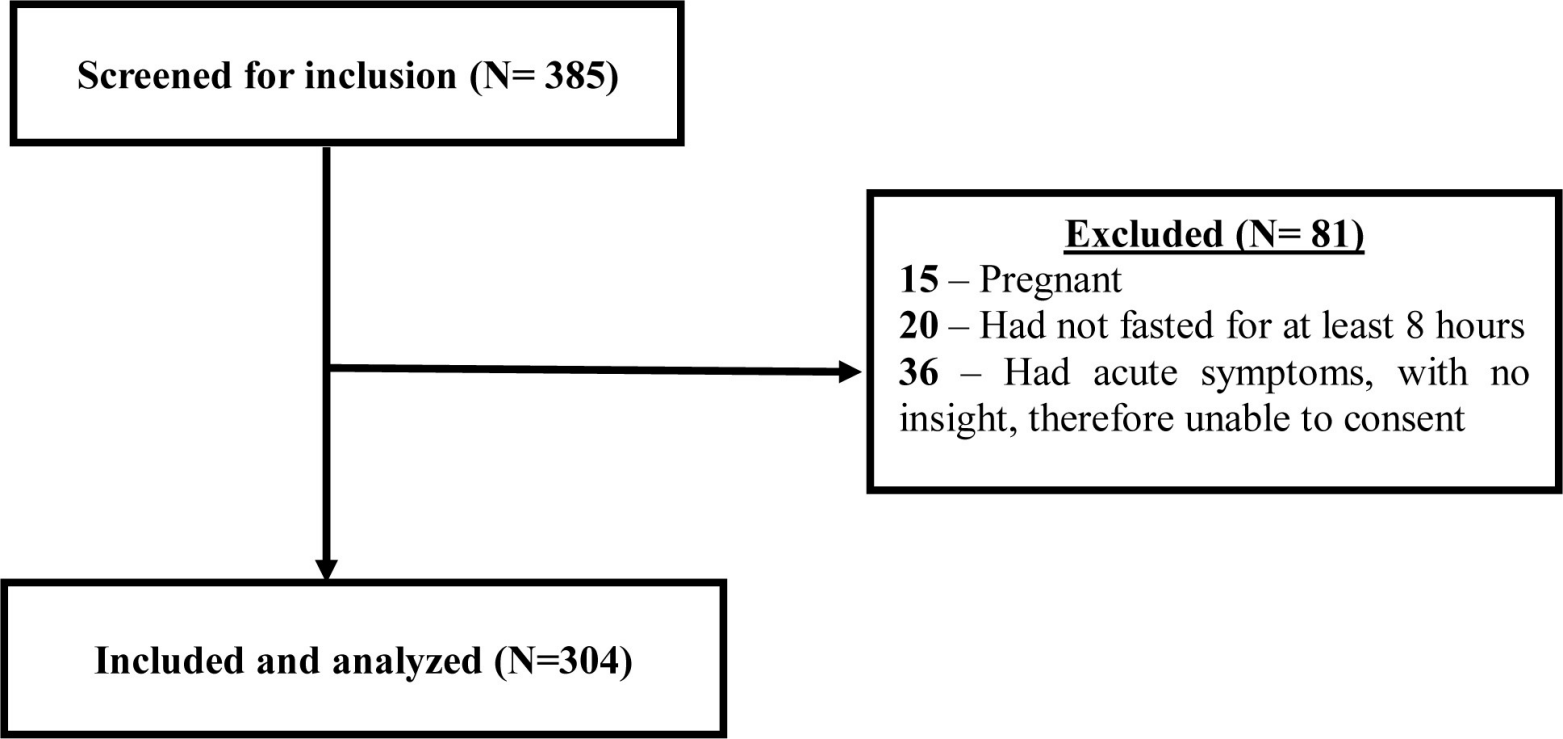
Flow chart for participants’ recruitment into the study.

Out of the 385 participants screened for inclusion into the study between October 2018 to march 2019, we present results of the 304 participants. We excluded 81 participants, for various reasons as shown in Fig 1.

Participants’ socio-demographic characteristics are presented in Table 1. The mean age of the participants was 38.56±13.66 years. The majority (39.8%) of the participants were married/living with a partner, had attained some level of education (88.82%), were employed (82.20%) and resided in rural areas (74.34%). Only 9.8% of the participants had ever smoked and 34.87% had ever taken alcohol. There were significantly more male smokers (p = 0.028) and alcohol drinkers (p<0.001) compared to females.

**Table 1:** Socio-demographic characteristics of the participants.

Characteristic	Overall (N = 304)	Male (N = 135)	Female (N = 169)	p-value
	n/N (%)	n/N (%)	n/N (%)	
**Age in years (mean±SD)**	38.56 ±13.66	37.2 ±13.55	39.65 ±13.69	0.120
**Marital status**				**<0.001**
Single	117 (38.49)	65 (48.15)	52(30.77)	
Married /Living with partner	121(39.80)	54 (40.00)	67 (39.64)	
Divorced/Separated/widow(er)	66 (21.71)	16 (11.85)	50 (29.59)	
**Education Level**				0.797
Never attended	34 (11.18)	14 (10.37)	20 (11.830	
≤Secondary	201 (66.12)	92 (68.15)	109 (64.50)	
Tertiary/University	69 (22.70)	29 (21.48)	40 (23.67)	
**Tribe**				0.865
Others	18 (5.92)	9 (6.67)	9 (5.33)	
Bakiga	21 (6.91)	10 (7.41)	11 (6.51)	
Banyankole	243 (79.93)	105 (77.78)	138 (81.66)	
Baganda	22 (7.24)	11 (8.15)	11 (6.51)	
**Religion**				0.940
Others	35 (11.51)	16 (11.85)	19 (11.24)	
Catholic	137 (45.07)	60 (44.4)	77 (45.56)	
Protestant	109 (35.86)	50 (37.04)	59 (34.91)	
Moslem	23 (7.57)	09 (6.67)	14 (8.28)	
**Employment**				0.105
Unemployed	45 (14.80)	15 (11.1)	30 (17.75)	
Employed	259 (82.20)	120 (88.89)	139 (82.25)	
**Area of residence**				0.924
Rural	226 (74.34)	100 (74.07)	126 (74.56)	
Urban	78 (25.66)	35 (25.93)	43 (25.44)	
**Ever Smoked**				**0.028**
No	274 (90.13)	116 (85.93)	158 (93.49)	
Yes	30 (9.87)	19 (14.07)	11 (6.51)	
**Ever taken Alcohol**				**<0.001**
No	198 (65.13)	65 (48.15)	133 (78.70)	
Yes	106 (34.87)	70 (51.85)	36 (21.30)	

The clinical characteristics of study participants are presented in Table 2. The mean weight of the participants was 68.18±14.14 kg. Males were significantly taller than females (p<0.001). The mean height for males was 1.68±0.07 m compared to 1.60±0.06 m for females. More than half (63.82%) of the participants had a diagnosis of bipolar disorder with 45.72% having been with mental illness for less than 5 years. Almost half (48.68%) of the patients had been on treatment for less than 5 years and most of the participants (81.91%) were taking antipsychotic medications with most of them (93.98) on typical antipsychotics. The majority (89.80%) had a low level of physical activity (< 150 min of moderate exercise or 75min of vigorous exercise per week). More females than males had a low level of physical activity (p = 0.046).

**Table 2:** Clinical characteristics of the participants.

Characteristic	Overall N = 304	Male (N = 135)	Female (N = 169)	p-value
	n/N (%)	n/N (%)	n/N (%)	
Weight, Kg (mean±SD)	68.18 ±14.14	67.13 ±12.97	69.03 ±14.99	0.244
Height, m (Mean±SD)	1.64 ±0.080	1.68 ±0.07	1.60 ±0.06	**<0.001**
**Level of physical activity**				**0.046**
< 150 min of moderate exercise or 75min of vigorous/ week	273 (89.80)	116 (85.93)	157 (92.90)	
≥150 min of moderate exercise or 75min of vigorous /week	31 (10.20)	19 (14.07)	12 (7.10)	
**Mental illness**				**0.046**
Bipolar disorder	194 (63.82)	86 (63.70)	108 (63.91)	
Schizophrenia	79 (25.99)	41 (30.37)	38 (22.49)	
Depression	31 (10.20)	8 (5.93)	23 (13.61)	
**Duration of Mental Illness (years)**				0.161
< 5	139 (45.72)	66 (48.89)	73 (43.20)	
5–10	69 (22.70)	34 (25.19)	35 (20.71)	
>10	96 (31.58)	35 (25.93)	61 (36.09)	
**Duration of Psychotropic Medication (years)**				0.177
< 5	148 (48.68)	69 (51.11)	79 (46.75)	
5–10	88 (28.95)	32 (23.70)	56 (33.14)	
>10	68 (22.37)	34 (25.19)	34 (20.12)	
**Participants on Antipsychotic medication**	249 (81.91)	113 (83.70)	136 (80.47)	0.467
**Antipsychotic class (N = 249)**				0.918
Typical	234 (93.98)	106 (93.81)	128 (94.12)	
Atypical	15 (6.02)	7 (6.19)	8 (5.88)	
**Mood stabilizer**	162 (53.29)	69 (51.11)	93 (55.03)	0.496
**Antidepressant**	56 (18.42)	20 (14.81)	36 (21.30)	0.147

The proportions of participants with different cardio-metabolic abnormalities are shown in Table 3. It should be noted that two of the participants did not undergo laboratory testing for lipid profile due to inadequate samples collected and therefore we do not present results of their fasting lipid profiles. Of all the participants, 46.38% were either overweight or obese with 33.22% having abdominal/central obesity. There were significantly more obese females compared to males (p<0.001) with 26.04% of all females obese compared to 9.63% of males. The proportion of participants with abnormal lipid profiles were as follows; 34.11% had low high-density lipoproteins, 37.42% had hypertriglyceridemia and 34.77% had hypercholesterolemia. The proportions of participants who had pre-diabetes and diabetes mellitus were 11.18% and 9.87% respectively and 40.46% of participants had hypertension.

**Table 3:** Cardio-metabolic abnormalities among study participants.

Characteristic	Overall N = 304	Male (N = 135)	Female (N = 169)	p-value
	n/N (%)	n/N (%)	n/N (%)	
Obesity based on BMI				**<0.001**
Overweight	84 (27.63)	32 (23.70)	52 (30.77)	
Obese	57 (18.75)	13 (9.63)	44 (26.04)	
Abdominal obesity	101 (33.22)	18 (13.33)	83 (49.11)	**<0.001**
Pre-Diabetes	34 (11.18)	16 (11.85)	18 (10.65)	0.943
Diabetes Mellitus	30 (9.87)	13 (9.63)	17 (10.06)	0.901
Hypertension	123 (40.46)	52 (38.52)	71 (42.01)	0.538
	**N = 302**	**N = 134**	**N = 168**	
Low HDL	103 (34.11)	40 (29.85)	63 (37.50)	0.164
Hypertriglyceridemia	113 (37.42)	50 (37.31)	63 (37.50)	0.973
Hypercholesterolemia	105 (34.77)	40 (29.85)	65 (38.69)	0.109

**BMI**: Body Mass Index, **HDL:** High-Density Lipoprotein.

## Discussion

Our results show high proportions of cardio-metabolic abnormalities among patients with SMI. According to our findings, one in three (33.22%) participants had abdominal obesity and two in five (46.38%) were either overweight or obese. This is slightly lower than the one in two (50%) that was found in two meta-analyses that reported the prevalence of cardio-metabolic abnormalities among patients with SMI [5, 6]. The proportion of participants with obesity in our study is much lower than that found in a study in North America which found a prevalence of obesity of almost 80% in a sample of more than 10,000 psychiatric outpatients with schizophrenia and bipolar disorder [23]. It is however comparable to the prevalence of obesity found in one meta-analysis of studies published between 1990 and 2010 which reported a prevalence of 45–55% among patients with schizophrenia and 21–49% among those with bipolar disorder [24]. The prevalence in our study was lower probably because the majority (93.98%) of the participants taking antipsychotics were on typical antipsychotics yet obesity among patients with SMI has been mainly associated with new generation (atypical) antipsychotics [8, 25]. In addition, 89.80% of all the participants had reduced levels of physical activity (<150 min of moderate exercise or 75min of vigorous exercise per week) yet reduced physical activity is a known risk factor for obesity. Based on BMI, 18.75% of all the participants were obese and this is 2.6 times higher than the prevalence of 7% found in a study among adults ≥ 18 years in Eastern Uganda [26] and is also higher than the global prevalence of 13% [27]. Our findings underscore the need for routine and systematic cardio-metabolic screening among patients with SMI in Uganda. Furthermore, there is need for interventions to address the high prevalence of obesity among patients with SMI. These interventions include: increase in physical activity, diet modification, careful selection of psychotropic medication for these patients based on their base-line laboratory findings; since some of the psychotropic medication such as atypical antipsychotic medication predispose patients to increased risk of obesity.

The proportion of participants with hypertension was 40.46%, which falls in the range found in a meta-analysis by De Hert and colleagues that found a prevalence of 19–58% among patients with schizophrenia and 35–61% among those with bipolar disorder [24]. This proportion is 1.5 times higher than the national prevalence of 26.4% in the general Ugandan population found in a nation-wide non-communicable disease (NCD) survey of 2014 [28] as well as the global prevalence of hypertension of 26% in the general population [29]. It is therefore important that patients with severe mental illness are screened for hypertension routinely during their reviews. This will enable early detection and timely management of hypertension in patients with SMI in Uganda.

One in three patients with SMI in this study had hypertriglyceridemia (37.42%) and hypercholesterolemia (34.77%) and about one in four (34.11%) had low levels of high-density lipoproteins (HDL). This is slightly lower than the 40% prevalence of hypertriglyceridemia found in the meta-analysis by Vancampfort and colleagues that described the prevalence of cardio-metabolic abnormalities among patients with SMI [6]. These proportions are however at least five times higher than those found in a community-based survey in southwestern Uganda in which hypercholesterolemia was at 6.0% and triglyceridemia at 5.0% [30]. This can be explained by the high levels of obesity and overweight (46.38%) in this population and the fact that some antipsychotic drugs affect lipid and triglyceride metabolism independent of weight gain [31]. Additionally, the unhealthy dietary habits and sedentary lifestyles of patients with SMI, predispose them to dyslipidemias [32]. Our findings, therefore call for interventions that are aimed at preventing/reducing the burden of dyslipidemias in the study population. These interventions include: carefull selection the psychotropic medications, dietary modifications and increased physical activity, among others.

Based on fasting blood sugar, 9.87% of all the participants had diabetes mellitus, a proportion three times higher than the 2.7% for urban areas and nine times higher than the 1.0% for rural areas of Uganda reported in a nation-wide non-communicable disease risk factor survey [33]. It is, however, lower than the global range of 10–15% among patients with SMI [34, 35]. Furthermore, our findings are in agreement with findings from other studies where the prevalence of DM in patients with schizophrenia and bipolar disorder was estimated to be two to three times [34, 35] or even four times [36, 37] higher than in the general population.

Overall, our findings demonstrate high prevalence of cardio-metabolic abnormalities among patients with SMI in southwestern Uganda. This emphasises the need for routine cardio-metabolic screening strategies in this patient population. Furthermore, there is need to incorporate medical care services into mental health care, as well as designing local and national guidelines for screening and management of cardio-metabolic abnormalities among patients with SMI.

Our study had limitations. First, the study was conducted in one hospital and findings may not be generalizable beyond the population of patients attending the mental health clinic at MRRH. Secondly, the study may have been subjected to social acceptability bias from participants wanting to provide socially desirable responses to questions about risky lifestyles of patients with severe mental illness such as smoking and alcohol consumption. Thirdly, the sample size was powered on the known prevalence of metabolic syndrome and not on the individual cardio-metabolic abnormalities making it difficult to confidently report the prevalence of the individual cardio-metabolic abnormalities.

## Conclusion

There is a high level of cardio-metabolic abnormalities among patients with severe mental illness at MRRH and thus metabolic screening for these abnormalities should be routinely done during psychiatric reviews. There is also need for national guidelines for screening of metabolic abnormalities among patients with SMI so that these abnormalities can be detected early enough at stages where they can either be reversed or delayed to progress to cardiovascular disease. There is a need to educate patients with SMI on lifestyle modification such as quitting smoking and alcohol consumption, exercising and diet modification. Caretakers of these patients should be involved in these health education talks so that they can provide social support to these patients.

## Supporting information

S1 Dataset (DTA)
