# Supplementary material for: Cardio-metabolic abnormalities among patients with severe mental illness at a Regional Referral Hospital in southwestern Uganda
Source: PLoS One. 2020 Jul 17;15(7):e0235956. doi: 10.1371/journal.pone.0235956 (PMC7367467; doi:10.1371/journal.pone.0235956)
Supplement: Attachment — Submitted filename: Response to Reviewers.docx [file pone.0235956.s002.docx]

**April 09^th^, 2020**

The Editor

PLOS ONE

Dear Editor,

We are pleased to learn that you consider publishing our manuscript titled ***“Cardio-metabolic abnormalities among patients with severe mental illness at a Regional Referral Hospital in southwestern Uganda”*** after revision as per the reviewers’ comments. Thank you for taking time to review our manuscript and for giving us very constructive comments that we believe are going to make our work better. We have addressed the reviewers’ comments and suggestions and feel that the paper is greatly improved and is now suitable for publication in ***PLOS ONE*.** A detailed response to each of the reviewers’ comments is enclosed.

Sincerely

Dr. David Collins Agaba

Mbarara University of Science and Technology

**RESPONSE TO REVIEWER’S COMMENTS**

**Major revisions**

**1. The authors state that the aim of this study is to describe the cardio-metabolic abnormalities among patients with SMI attending the specific clinic at MRRH. However, after reading the introduction, it is unclear why the authors want to**

**know these percentages; what do they want to do with these numbers? It is also unclear what the reference category is to which these numbers will be compared. Do the authors want to know the percentage of abnormalities in SMI patients as compared to the general population in Uganda, or as compared to SMI population in other countries? And why do the authors want to know this? The context of this study could be much more understandable if the authors explicitly state the underlying aim of why they want to know these percentages.**

**Response**: We have worked on the introduction to address this. We have added a paragraph that describes why we need to know the percentages of cardiometabolic abnormalities among patients with SMI. It reads “Although over 30% of Ugandans have some form of mental illness, there are no national or hospital-based screening guidelines for cardio-metabolic abnormalities among patients with SMI. Thus far, data on cardio-metabolic abnormalities among patients with SMI in Uganda are limited. The aim of this study was to describe the cardio-metabolic abnormalities among patients with SMI attending the outpatient mental health clinic at Mbarara Regional Referral Hospital (MRRH). Documenting the burden of cardio-metabolic abnormalities among patients with SMI is key, so as to inform policy makers to consider formulation of guidelines for screening of metabolic abnormalities. Routine and systematic cardiometabolic screening will enable initiation of appropriate management of cardio-metabolic abnormalities in a timely manner, in this patient population which will contribute towards improved quality of life. Moreover, there is epidemiological evidence to suggest that routine cardiometabolic screening could avert premature cardiometabolic disease among patients with SMI.” **(line 65-77)**

**2.** **For the reader, the procedure of including patients in the study is unclear. The manuscript does not provide any information about the procedure to inform patients about the study and to sign informed consent (should be described**

**in the method section, and not as one line in the ethics section). It is also unclear how many patients were eligible (i.e. how many patients were invited to participate), and how many patients refused to sign informed consent (drop-out;**

**should be described in the Results section). In addition, in the discussion, the authors mention the sample size calculation (line 236), which is not even described in the method section. This is all very important information that is**

**now lacking in the manuscript.**

Response:

The procedure for providing information about the study and to sign informed consent as well as details of sample size have been included: “We recruited participants consecutively as they came for review at the mental health clinic as long as they met the inclusion criteria until the sample size was obtained. The sample size of 304 was computed using the Kish Leslie formula of 1965 using 1.96 as the critical value at 5% level of significance, taking the prevalence of metabolic syndrome among patients with severe mental illness in sub-Saharan Africa to be 23.2%, based on a study from South Africa, 0.05 as a margin of error and adjusting for a non-response rate of 10%.

Participants who were eligible to participate in the study were given comprehensive information regarding the nature and purpose of the study and a consent document was read to them in the local language or english depending on the language they understood by a trained research assistant. They were given a chance to ask questions for clarity before they provided consent. Those who accepted to participate were given a consent form to sign.” (**Line 100-111) Materials & Methods section**

About the number of patients approached, those that declined or dropped out: A study flow chart has been included to address. Results section: **line 186-198**

A total of 385 participants were eligible but 81 were excluded (reasons listed in the flow chart) to remain with 304 who were recruited.

**3. In the method section (line 86), the authors mention that they recruited a total of 304 patients. This is a result and should therefore be in the results section.**

Response: This has been addressed. The statement has been corrected **(line 100)** and the number reported in results section.

**4. The authors state that the fasting blood sugar levels were obtained after eight hours of fasting. Is it realistic that patients had been fasting for eight hours? Or could the percentage of persons with pre-diabetes or diabetes be influenced by non-fasting blood sugar levels?**

Response: We made sure that all the participants had fasted for 8 hours. Those who had not fasted for the 8 hours were only included if they were willing to wait at the clinic till, they completed the 8 hours fast. This has been clarified in methods section**. Line 147-148.**

**5. In the results section, the authors give number for ‘smokers’, ‘for ever taken alcohol’, and for ‘physical activity’. It is unclear what questions were used to get these data; I believe this should be mentioned in the method section.**

Response: Thank you for this comment. We have improved this in the methods section under measurements **(line 124-128)**

**6. Table 2: did the authors only include persons with the diagnosis ‘Schizophrenia’, or were other psychotic diagnoses related to schizophrenia also included?**

Response: During participant recruitment, we only recruited participants with a confirmed diagnosis of schizophrenia and not other psychotic disorders since our inclusion criteria was SMI (bipolar, Major depression and schizophrenia)

**7. Table 2: the variables mentioned in the table are not clear. ‘Antipsychotic’ is not bold, and as a reader I do not have a clue what the authors mean with this. Please clarify. Also, in Table 2, the use of the heading ‘Duration of**

**psychotropic medication (yes)’, suggests that everyone uses psychotropic medication. After calculating the total numbers, is seems indeed that everyone is using medication. But, why are ‘antipsychotics’ framed as a separate variable and why are the types of medication for depression or bipolar disorder not given? The order of the table and the missing information leaves the reader with questions, please adjust the table.**

Response: Table 2 has been improved to bolden “antipsychotic” but has also been improved to **“participants on antipsychotic medication”**

It is true everyone was on psychotropic medication but not everyone was taking an antipsychotic medication. To our understanding, psychotropic medication includes antipsychotics, mood stabilizers and antidepressants. We give details of the class of antipsychotic medication and not for mood stabilizers or anti-depressants: This is informed by literature that shows differences in causation of cardiometabolic abnormalities among patients with SMI according to class of antipsychotic medication yet we don’t find any special considerations for mood stabilizers & antidepressants in literature.

**8. Discussion: because for the reader, it is unclear why the authors would like to study these percentages (what the bigger aim is of knowing these numbers?), the discussion reads like a story summing up percentages without truly**

**delivering a message. The percentages from the study are compared to percentages in either other SMI populations and to percentages in the general population of Uganda. I think the authors could truly improve the discussion by**

**making clear what they want to do with these numbers, and to whom they want to compare these numbers, so that they can come up with a more in-depth discussion what could be done with these numbers. Now, the discussion only**

**represents four paragraphs in which the numbers are compared to others, without drawing a conclusion. I think only two paragraphs should be spend on comparing these numbers, and thereafter, the authors should focus on the wider**

**picture. Also, the phrase ‘unhealthy lifestyles’ pops up now and then, it is slightly touched, but I think the authors have more ideas about the role of unhealthy lifestyles and the early detection of cardio-metabolic abnormalities in solving the problem of these high cardio-metabolic abnormalities.**

Response: Thank you for the guidance. We have tried to improve the discussion to make sense out of the numbers and we hope it reads better now.

For example:

I. “Our findings underscore the need for routine and systematic cardio-metabolic screening among patients with SMI in Uganda. Furthermore, there is need for interventions to address the high prevalence of obesity among patients with SMI. These interventions include: increase in physical activity, diet modification, careful selection of psychotropic medication for these patients based on their base-line laboratory findings; since some of the psychotropic medication such as atypical antipsychotic medication predispose patients to increased risk of obesity.” **Line 252-259**
II. “Overall, our findings demonstrate high prevalence of cardio-metabolic abnormalities among patients with SMI in southwestern Uganda. This emphasises the need for routine cardiometabolic screening strategies in this patient population. Furthermore, there is need to incorporate medical care services into mental health care, as well as designing local and national guidelines for screening and management of cardiometabolic abnormalities among patients with SMI.” **Line 292-297**

**9. The conclusion summaries actually all that the reader wants to know more of in the introduction and the discussion. I think the conclusion has a very clear message of the why the authors want to know these numbers and what they aim to do after knowing these numbers. I think that the message of the conclusion should be highlighted more in the introduction (why do you want to know this) and in the discussion (now you know this, what can you do**

**With this information).**

Response: Thank you for this comment and for your guidance on how best to improve our paper. The suggestions have been included in the introduction and discussion sections.

**Minor revisions**

**1. Please be consistent in the use of the word cardio-metabolic (cardio-metabolic vs cardiometabolic).**

Response: The word has been changed to cardio-metabolic across the entire document.

**2. The sentence in the abstract from line 16 to 20 is unreadable, please rephrase.**

Response: The sentence has been rephrased. Hope it reads better. “Although over 30% of Ugandans have some form of mental illness, there are no national or hospital-based screening guidelines for cardio-metabolic abnormalities among these patients, a general trend in most low-income countries. The screening rates for cardio-metabolic abnormalities in most low-income countries are at only 0.6%.” **Line 17- 21**

**3. The author describes in line 46 to 50 the known percentages of cardio-metabolic abnormalities in SMI patients found in two previous meta-analyses. For the reader it’s unclear why the percentage of smokers is included in this**

**summary, since this is a lifestyle behaviour, not a cardio-metabolic abnormality.**

Response: The statement has been improved. The percentage of smokers has been eliminated because it is not a cardio-metabolic abnormality. **(Line 52-54)**

**4. The Introduction reads as one large paragraph. I believe that the use of more paragraphs would provide a better structure of the paper. The author could for example start a new paragraph from line 54. Also, the method section**

**could use some paragraphs.**

Response: we have improved the sections by creating more paragraphs in the introduction and methods section.

**5. In line 59, for the reader it’s unclear to what the word ‘they’ refers; it mostly reads as it refers to ‘lifestyle modification approaches’, but this is probably not what is meant. Please clarify in the text.**

Response:

The statement has been improved to: “The low screening rates result in late diagnosis of these abnormalities at stages where lifestyle modification approaches are no longer very beneficial, thus leading to progression to cardiovascular disease.” **Line 62-64**

**6. It is unclear what is meant with ‘acute symptoms with no insight’ (line 80). Please rephrase.**

Response: The statement has been rephrased to “We excluded all patients who had acute symptoms of mental illness, those who had no insight and those considered unable to consent by the attending clinician” **Line 95-97**

**7. The words ‘history of smoking’ are mentioned twice in the summary (line 90 and 91).**

Response: This has been corrected. The repeated word has been deleted.

**8. When starting a new paragraph, it’s not preferable to start the first sentence with ‘These’ since the reader is unaware to what the word ‘these’ refers (line 112).**

Response: This has been corrected. (line 146)

**9. Line 117: please do not use a capital for the word Triglycerides**

Response: we have written the word properly as triglycerides (line 152)

**10. Please delete the word ‘obese’ in line 128, as it is redundant.**

Response: The word has been removed.

**11. In my opinion, the sentence about data collection (line 145) should be in de method section, not in the ethics section.**

Response: The sentence has been moved to methods section **(Line 120-121)**

**12. In the tables, I believe it’s preferable to make the significant p-values bold, so it’s easier to scan the table.**

Response: the significant p-values have been made bold in all tables.

**13. In the tables, I would prefer to see the mean ± standard deviation not including ( ).**

Response: This has been corrected in all tables and in the text.

**14. In Table 1, the Overall numbers for Single persons do miss a ) . I think the 0 should be deleted and replaced by a), also to keep the number of decimals equal in the table. In Table 1, also, a space between ‘Female(N=169)’ is needed.**

Response: Table 1 has been corrected as per the suggestions

**15. The marital status ‘died’ is a bit odd; I think ‘widow(er)’ would be more appropriate.**

Response: This has been corrected in table 1

**16. Please replace the ‘&’ with ‘and’ throughout the whole manuscript**

Response: The correction has been made all through the document.

**17. Please write numbers below ten as a word instead of a number, e.g., ‘3 psychiatrists’ should be ‘three psychiatrists’**

Response: the changes have been made all through the document. For example: **line 84-86**

**18. Please be consistent in the use of a space between the number and the percentage-sign throughout the whole**

**manuscript (e.g. either 10% or 10 %).**

Response: The change to use of no space has been effected all through the document

**19. Describe square meters using the superscript function (i.e. m2)**

Response: This has been corrected. Line: 151-153

**20. Line 192: it’s unclear where the word ‘that’ refers to, please clarify**

Response: This has been corrected to: “the prevalence of obesity” **Line 242**
